# A stable genetic polymorphism underpinning microbial syntrophy

**DOI:** 10.1038/ismej.2016.80

**Published:** 2016-06-03

**Authors:** Tobias Großkopf, Simone Zenobi, Mark Alston, Leighton Folkes, David Swarbreck, Orkun S Soyer

**Affiliations:** 1 School of Life Sciences, The University of Warwick, Coventry, UK; 2 The Genome Analysis Centre, Norwich Research Park, Norwich, UK

## Abstract

Syntrophies are metabolic cooperations, whereby two organisms co-metabolize a substrate in an interdependent manner. Many of the observed natural syntrophic interactions are mandatory in the absence of strong electron acceptors, such that one species in the syntrophy has to assume the role of electron sink for the other. While this presents an ecological setting for syntrophy to be beneficial, the potential genetic drivers of syntrophy remain unknown to date. Here, we show that the syntrophic sulfate-reducing species *Desulfovibrio vulgaris* displays a stable genetic polymorphism, where only a specific genotype is able to engage in syntrophy with the hydrogenotrophic methanogen *Methanococcus maripaludis*. This 'syntrophic' genotype is characterized by two genetic alterations, one of which is an in-frame deletion in the gene encoding for the ion-translocating subunit *cooK* of the membrane-bound COO hydrogenase. We show that this genotype presents a specific physiology, in which reshaping of energy conservation in the lactate oxidation pathway enables it to produce sufficient intermediate hydrogen for sustained *M. maripaludis* growth and thus, syntrophy. To our knowledge, these findings provide for the first time a genetic basis for syntrophy in nature and bring us closer to the rational engineering of syntrophy in synthetic microbial communities.

## Introduction

Syntrophic interactions represent cases of metabolic cooperation between two phenotypically distinct organisms (Schink, 1997; McInerney *et al.*, 2008; Sieber *et al.*, 2012; Morris *et al.*, 2013). These interactions are common among microbes living in environments that can be readily depleted of strong electron acceptors. In such environments, including anaerobic reactors, animal guts, ocean and lake sediments and soil, the depletion of suitable electron acceptors is expected to increase the abundance of microbes with fermentative metabolism. The low thermodynamic energy associated with fermentative metabolic pathways results in 'thermodynamic inhibition' of microbial growth due to product accumulation (Schink, 1997; Kleerebezem and Stams, 2000; Großkopf and Soyer, 2014, 2016). This inhibition of growth in the primary degrading microbes can be lifted by others consuming the inhibitory waste product (mostly hydrogen) (Seitz *et al.*, 1988; Schink, 1997; Sieber *et al.*, 2012). Thus, environmental depletion of electron acceptors provides a setting in which syntrophic interactions can more readily emerge. Besides their central role in natural microbial communities (McInerney *et al.*, 2009; Sieber *et al.*, 2012), syntrophic interactions also constitute a desired motif in engineered synthetic microbial communities, where they can provide a metabolic basis for stable community formation (Gilbert *et al.*, 2003; Großkopf and Soyer, 2014; Santala *et al.*, 2014).

Despite their importance in ecological and engineered settings, our understanding of the molecular basis of syntrophic interactions remains limited. Transcriptomic analyses in a syntrophic model system (Walker *et al.*, 2012, 2009) have highlighted several genes that are differentially regulated under conditions of syntrophy. The gene products are involved in primary energy metabolism as well as in secretion of secondary metabolites which are suggested to enhance the growth of the syntrophic partner (Walker *et al.*, 2009, 2012). Other studies indicated genes involved in formation of spatial structures to be important for syntophic interactions (Schink, 1997; Kato and Watanabe, 2010; Summers *et al.*, 2010). Indeed, biofilm or granule formation (Summers *et al.*, 2010; Wintermute and Silver, 2010a; Worm *et al.*, 2014), as well as direct attachment via flagella (Marx, 2009; Shimoyama *et al.*, 2009; Kato and Watanabe, 2010), is observed in several co-cultures involving syntrophy or cross-feeding. However, formation of spatial structures like biofilms is also frequently observed in monocultures (Zhang *et al.*, 2007) and under non-syntrophic conditions (Nadell *et al.*, 2009), and as such, it is not clear whether biofilm formation is a key driver in the formation and maintenance of syntrophies.

At a cellular level, it is well documented that syntrophic associations result in transcriptomic and metabolomic changes in the involved organisms (Walker *et al.*, 2009, 2012; Qi *et al.*, 2014). The study of these changes has led to the identification of specific genes, whose deletion completely abolishes the ability to form syntrophic interactions (Walker *et al.*, 2009). Despite these important insights, it is still not clear whether there are specific genetic alterations that can enhance formation of syntrophies. If such alterations exist, then identifying these could allow for a better understanding of the ecological and evolutionary basis of natural syntrophies and enable engineering of synthetic ones. The latter objective is motivated by observations that synthetic engineering of syntrophic and mutualistic interactions among microbes can increase bioproductivity (Shou *et al.*, 2007; Wintermute and Silver, 2010b; Kerner *et al.*, 2012; Mee *et al.*, 2014) and scope of biotechnological applications (Gilbert *et al.*, 2003; Bernstein *et al.*, 2012; Santala *et al.*, 2014).

Here, we aim to identify genetic drivers of syntrophy by focusing on physiological and genetic analysis of the well-established model syntrophic system between a sulfate-reducing bacteria (SRB) *Desulfovibrio vulgaris* strain Hildenborough (DvH) and a hydrogenotrophic methanogen *Methanococcus maripaludis* S2 (Mm) (Stolyar *et al.*, 2007; Hillesland and Stahl, 2010). A typical SRB, DvH is equipped with a dissimilatory sulfate reductase and various hydrogenases (Pieulle *et al.*, 2005), which allows it to utilize hydrogen, while reducing sulfate. The ability to utilize hydrogen makes SRB a competitor for methanogens like Mm in the presence of sulfate. In the absence of sulfate, however, its fermentative metabolism results in the production of hydrogen and opens up the potential of a syntrophic interaction with Mm.

We show that this ability to engage in syntrophy with Mm is underpinned by a stable genetic polymorphism in DvH; under a sulfate-free, minimal lactate media, only a specific genotype of DvH can grow syntrophically with Mm. We find that this genotype naturally exists and is characterized by a set of two distinguishing genetic mutations. Both mutations are linked to the lactate oxidation pathway, the energetics of which is a key driver of the syntrophic interaction. In particular, the resulting altered thermodynamics of this pathway increases the tolerated hydrogen pressure of lactate oxidation in DvH, therefore providing substrate for growth of Mm and a stable syntrophy can be formed. These findings provide the first evidence of a specific syntrophy-inducing genetic polymorphism in SRB and point toward energetic determinants of metabolism as a key driver for syntrophy formation in general.

## Materials and methods

### Culturing and media

Cultures of *Desulfovibrio vulgaris* strain Hildenborough (DSM644, DvH) and *Methanococcus maripaludis* S2 (DSM2067, Mm) were ordered from DSMZ (www.dsmz.de). Cultures of Mm were grown in medium 141, as specified on the DSMZ homepage, or in Co-Culture Medium (CCM) (Walker *et al.*, 2009) with slight modifications (for details, see Supplementary Methods). Cultures of DvH were grown in Postgate medium C (Postgate, 1984) (for DNA extraction and plating) or in CCM. Co-cultures of DvH and Mm were grown in CCM. Initial co-cultures were started by injecting 1/20 volume of each CCM-washed monoculture into fresh CCM. Culturing conditions were 37 °C in a static incubator (Heratherm, Thermo Scientific, Waltham, MA, USA) in the dark unless otherwise specified. The culture vessels used were either anaerobe tubes (Chemglass Life Sciences, Vineland, NJ, USA) or 50 and 100 ml serum flasks (Supelco, Bellefonte, PA, USA) with blue butyl rubber stoppers (Chemglass Life Sciences). Subculturing was performed by injecting 1/20 of the volume of a late log-phase culture into fresh culture medium (for example, 250 μl into 5 ml). The detailed recipes and preparation protocols of the media used are supplied in the Supplementary Methods.

### Optical density and gas production

Optical density (OD) was measured at 600 nm in a spectrophotometer (Thermo Scientific). Gas measurements were performed on an Agilent 7890 A Gas chromatograph (Agilent Technologies, Craven Arms, UK). For detailed procedures and calculations for both OD and gas measurements, see Supplementary Methods.

### Microscopy

For microscopic analysis, 5 μl of culture was placed on a microscope glass slide and covered with a glass coverslip. The coverslip was sealed to the slide with transparent nail-varnish to minimize evaporation. Slides were placed on an inverted fluorescence microscope (IX83, Olympus, Tokyo, Japan) and observed under × 600 magnification in phase contrast or using a blue light excitation at 440 nm generated by a pE-2 LED system (CoolLed, Andover, MA, USA) and a band-pass CFP filter at 503–538 nm (Olympus). Mm has a co-factor (F_420_) that shows blue-green fluorescence upon illumination with blue light (Peak Ex/Em=420/470 nm) (Doddema and Vogels, 1978). Images were recorded in black and white on a CoolSnap HQ^2^ camera (Photometrics, Tucson, AZ, USA) and false (blue) color added to the fluorescent channel image. Image overlays were generated in the camera software cellSens Dimensions (Olympus) and exported as image files.

### DNA extraction

For DNA extraction, 30 ml of DvH cultures was grown in liquid culture (Medium C) until late log-phase. Cells were harvested by centrifugation at 12 300 *g* for 3 min in a tabletop centrifuge (Microfuge SCF2, Stuart, Stone, UK) and the resulting pellet frozen at −20 °C until DNA extraction using the Wizard gDNA purification kit for Gram-negative bacteria (Promega, Fitchburg, WI, USA). Extraction resulted in 110–200 μg of DNA per sample. This still was below the purity requirements for Illumina sequencing as confirmed by 260/280 nm and 260/230 nm absorbtion ratio measurements (~1.8 and ~1.4, respectively) on a CLARIOstar plate reader (BMG Labtech, Ortenberg, Germany) equipped with LVis plate (BMG Labtech). For further purification, extracted DNA was bound to a purification column of the PureLink Genomic DNA Mini Kit (Invitrogen, Carlsbad, CA, USA) and eluded in buffer. In detail, 40 μl of extracted gDNA samples was treated with the following steps: add 200 μl milliq-H_2_O, add 200 μl PureLink GenomicLysis/Binding Buffer, vortex at 2200 r.p.m. for 5 s, add 200 μl ethanol (100%), vortex for 5 s. The resulting 640-μl mixture was loaded to a PureLink Spin Column and from there on, the manufacturer's instructions for DNA purification were followed. The resulting DNA quantity was about 1/6 of the original sample (~10 μg per sample); however, the 260/280 nm and 260/230 nm ratios were around 1.8 and 2.2, respectively, which indicates high purity DNA samples.

### Library preparation and MiSeq sequencing

Illumina TruSeq DNA libraries were prepared at The Genome Analysis Centre (TGAC, Norwich, UK) following the manufacturer's protocol (15036187; Illumina, San Diego, CA, USA) with some minor modifications. In brief, samples from DvH-s and DvH-ns clones were normalized to 1 μg of input DNA and sheared by sonication via a Covaris S2 to ~500 bp long fragments. Following ligation of indexing adapters to the DNA fragments, samples were amplified by PCR. The insert size of the DNA libraries was verified on a PerkinElmer GX using the High Sensitivity DNA chip and the concentration determined by a High Sensitivity Qubit assay. Beckman Coulter XP beads (Part No: A63880) were used for size selection. The resulting 10 genomic DNA paired-end libraries were pooled, spiked with 1% PhiX Control v3 and sequenced with 150 bp paired-end run metrics on the Illumina MiSeq platform (MiSeq Reagent Kit v2, using MiSeq Control Software 2.5.0 and RTA 1.18.54). The fastq data files were deposited in the European Nucleotide Archive and may be accessed at http://www.ebi.ac.uk/ena/data/view/PRJEB12162.

### Variant calling

The reads from each sample were quality trimmed using Sickle (Joshi and Fass, 2011) and mapped independently to the DvH reference genome (http://bacteria.ensembl.org/desulfovibrio_vulgaris_str_hildenborough/Info/Index/, accessed 9/2/15) using the sequence aligner BWA-SW v0.7.12 (Li and Durbin, 2010). The resulting alignments were converted into binary format and position sorted using SAMtools v1.1 (Li *et al.*, 2009). To call single-nucleotide polymorphisms (SNPs) and insertions/deletions (INDELS), SAMtools' mpileup was used on the processed alignments and the output passed onto VarScan v2.3.7 which was run with default settings (Koboldt *et al.*, 2012). The effects of the called variants within the resulting VCF files produced by VarScan were annotated using SnpEff v4.0 (Cingolani *et al.*, 2012).

### DvH core metabolic pathways and their thermodynamic representation

A representation of the core metabolic (catabolic) system of DvH (as shown in Figure 3) was produced based on information from the literature (Stolyar *et al.*, 2007; Walker *et al.*, 2009) and bioinformatics databases Rhea (http://www.rhea-db.org/home) (Morgat *et al.*, 2015), ChEBI (https://www.ebi.ac.uk/chebi/) (Hastings *et al.*, 2013) and IMG (https://img.jgi.doe.gov/cgi-bin/m/main.cgi) (Markowitz *et al.*, 2012). The full chemical formula for each reaction, as well as their thermodynamic data, is given in Supplementary Table S1, while chemical abbrevations used are further described in Figure 3 legend and Supplementary Table S2. The compiled thermodynamic values listed in Supplementary Table S1 are taken from the literature (Thauer *et al.*, 1977) and used to compute redox potentials for electron accepting or donating reactions as shown in Figure 3. The downward shift in redox potential of Coo in Figure 3 is calculated based on a transport of two single charged ions per hydrogen produced over a 100-fold concentration gradient across the membrane (~−23 kJ mol^−1^).

## Results and discussion

### DvH-Mm syntrophy requires a specific DvH genotype

To study the potential genetic basis of syntrophic interactions, we used here the model system of DvH and Mm. In the absence of sulfate, and under minimal, lactate-containing media, the DvH-Mm co-culture has been shown to syntrophically oxidize lactate to produce methane, acetate and CO_2_ while relying mainly on hydrogen exchange between the two species (Stolyar *et al.*, 2007). When we attempted to initialize this system, the initial frequency of co-culture success, assessed with methane formation and OD measurements, was only 1/40 (Supplementary Figure S1a). This observation can be explained by the presence of a low frequency phenotypic or genetic variant in the wild-type population of the DvH that is characterized by its ability to form a successful syntrophy with Mm. In the case of phenotypic variability, where a single genotypic background gives rise to multiple phenotypes (Balaban *et al.*, 2004), it would be expected that DvH clones isolated from the working syntrophic co-culture and re-cultured with Mm should again lead to low frequency in co-culture success. In contrast, in the case of genotypic variability, we would expect DvH clones isolated from the working syntrophic co-culture to give much higher co-culture success when re-cultured with the Mm.

**Figure 1:**
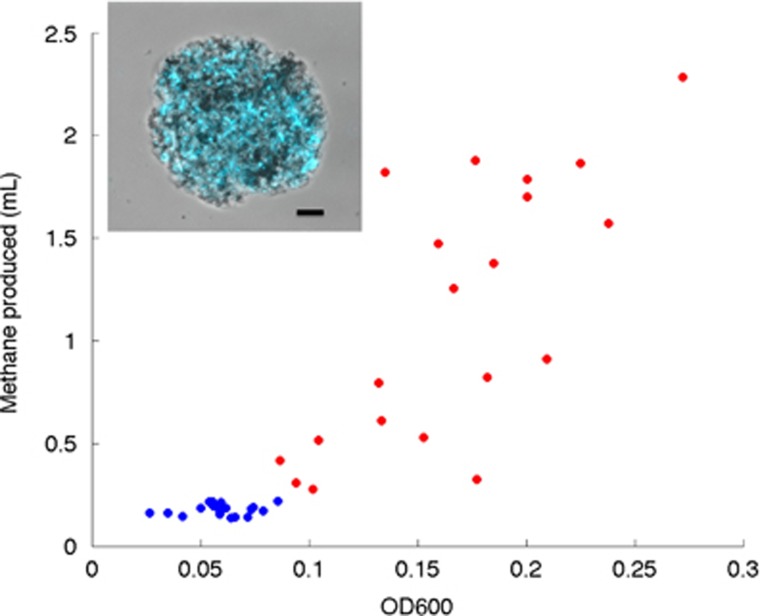
OD at 600 nm (OD600) and methane production of DvH-ns/Mm (blue, 20 replicates) and DvH-s/Mm co-cultures (red, 20 replicates) grown on CCM (*see Materials and methods*). The inset shows a sample, phase-contrast image of a granule from a DvH-s/Mm co-culture taken at × 600 magnification, and overlayed with the emission from the blue fluorescence channel. Scale bar=10 μm. The blue autofluorescence originates from the F_420_ cofactor of the methanogen.

To test these possibilities, we isolated 20 clones of DvH both from the working syntrophic co-culture and from the original inoculum culture of DvH. Re-inoculating these 40 DvH clones with Mm (see Materials and methods, Supplementary Figure S1b), we found that all co-cultures involving clones isolated from the working syntrophic co-culture led to increased OD and methane production after 1 week of growth (Figure 1). For the 20 co-cultures started with clones from the working syntrophic co-culture, the average methane production after 1 week was 1.13 ml (±0.64 ml, *n*=20), while it was 0.18 ml (±0.03 ml, *n*=20) for the co-cultures started with clones from the original DvH population. The pattern was consistent after subculturing (1/20 inoculum), with the second generation of cultures producing 1.03 ml (±0.46 ml, *n*=20) and 0.07 ml (±0.05 ml, *n*=20) methane for DvH clones isolated from the working syntophic co-culture and the original culture, respectively. For the successful co-cultures, we also observed a positive correlation between the methane production and OD (*r*^2^=0.54, V(ml)_CH4_=5.23 × OD−0.44); however, the latter is not a good predictor of growth due to a high degree of granule formation in the co-culture and associated patchiness (see Figure 1, inset). The consistent co-culturing success of DvH clones isolated from the working syntrophic co-culture leads to the conclusion that the observed success in formation of syntrophy is due to a genetic factor and not due to phenotypic variability. Hereafter, we refer to the clone leading to syntrophic co-cultures with Mm as 'DvH-s' and to the other clone as 'DvH-ns', for ‘non-syntrophic'.

### Genetic analysis of DvH-s vs DvH-ns reveals determinants of syntrophy

**Figure 2:**
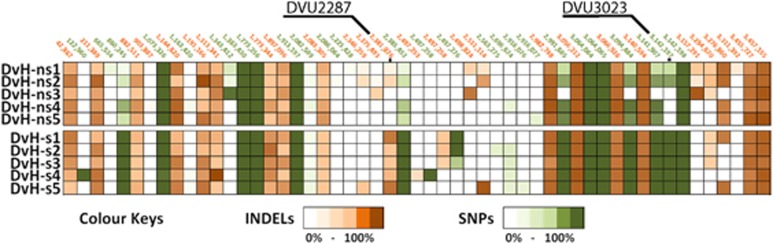
Mutations identified on the genome of 10 clones isolated from a working syntrophic co-culture (clones initially labeled as DvH-s 1–5) and from the wild-type DvH population (clones initially labeled as DvH-ns 1–5). Each square represents an SNP (green) or INDEL (brown), as identified by comparing the genome sequence of the corresponding clone with the reference DvH genome. The color scale of each square encodes the proportion of reads that contain the corresponding SNP or INDEL. The genomic location of each mutation is shown at the top. In total, 26 SNPs and 27 INDELs were identified on the chromosome. An additional two SNPs and two INDELs were identified on the megaplasmid that are not shown in this figure (but included in the Supplementary File 1). As discussed in the main text, 2 out of the 57 mutations were present in all seven ‘co-culturable' clones (DvH-s 1–5, DvH-ns 1 and DvH-ns2), a synonymous point mutation in the gene DVU3023 and a disruptive INDEL in the gene DVU2287.

To better understand the genetic difference between these two genotypes, we isolated five new clones of DvH-s from a successful co-culture and five clones from the original DvH population, which were expected to be DvH-ns. We re-sequenced the genomes of all of these 10 clones and annotated each genome against the reference DvH genome, which contains the main chromosome and a megaplasmid (Heidelberg *et al.*, 2004) (see *Materials and methods*). We found a total of 57 polymorphisms across the 10 clones. There were a total of 26 SNPs on the chromosome (Figure 2) and 2 on the megaplasmid, as well as 27 INDELs on the chromosome (Figure 2) and 2 more on the megaplasmid. A detailed view of the genotype of each strain for all 57 positions is provided in Supplementary File 1.

**Figure 3:**
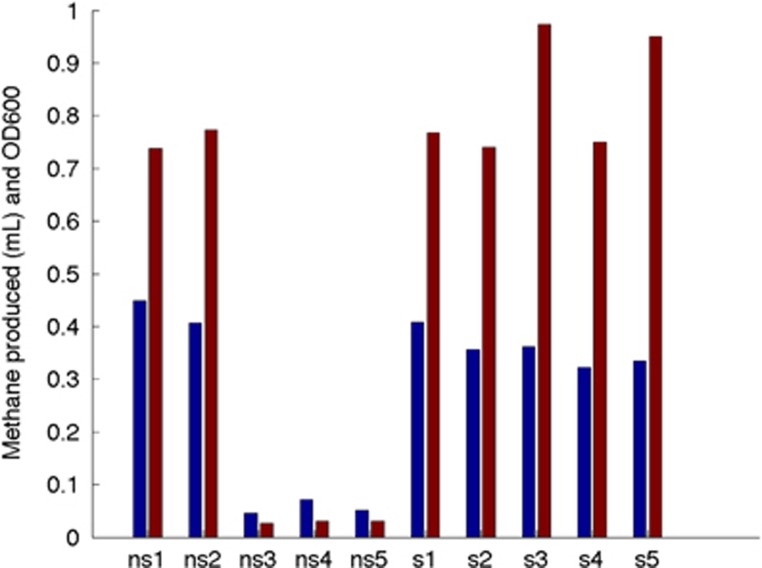
OD600 (blue) and methane production (red) of five DvH-ns and DvH-s clones respectively, when grown in co-culture with Mm in CCM (see Materials and methods). A full color version of this figure is available at the *The ISMEJ Journal* journal online.

To verify again the ability of each of these sequenced clones to form a successful syntrophy or not, we co-cultured them with Mm. These experiments revealed that as expected all five DvH-s clones (labeled as DvH-s 1–5) were able to generate a methane producing, syntrophic co-culture with Mm (Figure 3). Among the DvH-ns clones, three resulted in no co-culture growth as expected, while two (initially labeled as DvH-ns 1 and DvH-ns2) did result in relatively successful syntrophic co-culture (Figure 3) (these findings are further discussed below). Taking these physiological results in accord, we analyzed the sequence data for genetic changes that are common to all seven clones that led to successful co-cultures, and that are lacking from the other three clones. Out of all 57 mutations, only 2 were present in all 7 ‘co-culturable' clones, but absent in clones DvH-ns 3–5 (Figure 2), suggesting that these two mutations are essential for syntrophy. The first of these two mutations is a non-synonymous point mutation from G to A in the gene DVU3023 (DvH chromosome location: 3142197), resulting in an amino-acid exchange from Aspartate to Asparagine. The DVU3023 gene is annotated to encode for a sigma-54-dependent response regulator that controls the downstream operon (DVU3025–DVU3033), which includes the genes lactate permease and pyruvate decarboxylase. Notably, both enzymes are involved in the lactate oxidation pathway, with the first enzyme catalyzing the lactate uptake and the second catalyzing conversion of the pyruvate resulting from lactate oxidation. The second mutation is an in-frame deletion that led to the removal of a GAG sequence in the gene DVU2287 (DvH chromosome location: 2381876), resulting in the replacement of a TGA codon with a TCG codon. DVU2287 is annotated to encode for the CooK subunit of Coo, which is annotated as the H^+^/Na^+^ ion-translocating subunit of the carbon monooxide-dependent, membrane-bound dehydrogenase, which is hypothesized to be involved in the lactate oxidation pathway (Walker *et al.*, 2009). The TGA codon is known to encode for a stop codon, which can either lead to a truncated gene product or introduction of a Selenocysteine residue during translation (Valente *et al.*, 2005). Thus, in all seven genotypes able to form syntrophy, this replacement of the TGA codon with the TCG codon either resulted in a gain-of-function at the DVU2287 gene or to an exchange of a Selenocysteine residue with a Serine residue. Interestingly, sequencing data revealed that clones DvH-ns 1 and DvH-ns2, which were picked from the original DvH population but could form syntrophy with Mm, show a mixed signature of alleles consisting of the mutated and non-mutated sequences at this locus (Figure 2). This could be explained by the initial clone picking resulting in a heterogeneous sample of genotypes, or by the presence of multiple genome copies within one cell—a case that is known to be common in SRB (Postgate *et al.*, 1984).

### A thermodynamic model highlights potential implications of the *cooK* mutation

**Figure 4:**
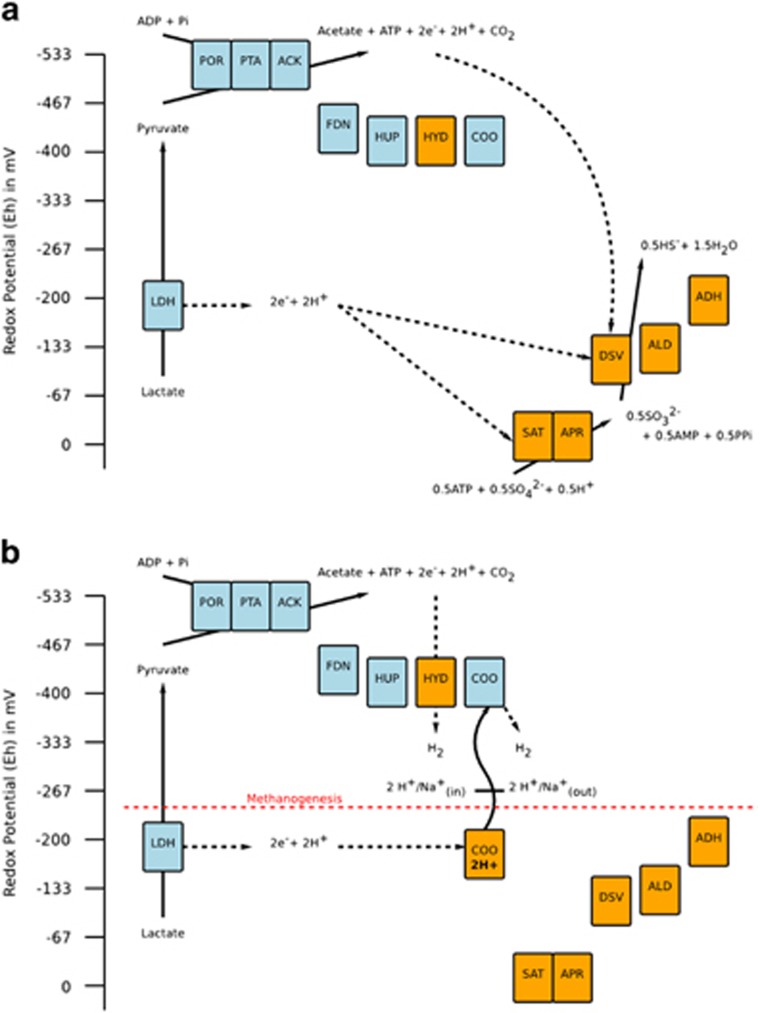
A cartoon representation of the lactate oxidation and sulfate respiration pathways in DvH. Each of the enzymes involved in these pathways is placed on the cartoon according to the standard reduction potentials of the redox reactions that they catalyze (see Supplementary Table S1). In the lactate pathway, lactate is first oxidized to pyruvate and then to acetate, each reaction releasing two electrons and two protons. The protons can then be reduced to produce hydrogen, in a reaction catalyzed by the different hydrogenases. The enzymes catalyzing electron releasing and accepting reactions are shown in blue and orange, respectively (enzymes that are connected represent combined reactions). The solid and dashed arrows represent the flow of matter and electrons, respectively. Note that due to thermodynamics, electrons can only flow from reactions with more negative reduction potentials to reactions with more positive reduction potentials. (**a**) In the presence of sulfate, lactate oxidation can be coupled to the electron accepting reactions of the sulfate pathway. (**b**) In the absence of sulfate, it is predicted that electrons from lactate oxidation flow to proton reduction mediated by Coo. The reduction potential of that reaction is predicted to be shifted downwards by energy investments from translocating two sodium/proton across a membrane gradient. The added energy allows an equilibrium hydrogen pressure equal to the other hydrogenases, acting as an elevator for electrons (the curved arrow). The horizontal dashed red line is the redox potential at which hydrogentrophic methanogens are assumed to accept electrons (−244 mV), and is included as a reference point. The enzyme labels are LDH=lactate dehydrogenase, POR=pyruvate-ferredoxin oxidoreductase, PTA=phosphate acetyltransferase, ACK=acetate kinase, FDN=formate dehydrogenase, HUP=uptake hydrogenase, HYD=release hydrogenase, COO=Coo hydrogenase (potentially H^+^/Na^+^ dislocating), SAT=ATP-sulfate adenylyltransferase, APR=adenylyl sulfate reductase, DSV=sulfite reductase, ALD=alanine dehydrogenase, ADH=ethanol dehydrogenase.

Given the well-established role of thermodynamic limitations as the basis of syntrophic interactions and the role of hydrogen formation in this (Cord-Ruwisch *et al.*, 1988; Schink, 1997; Noguera *et al.*, 1998), it is possible that the identified mutation in the Coo hydrogenase relates to the thermodynamics of the lactate oxidation pathway. To explore this possibility, we performed a thermodynamic analysis of this pathway as it is seen in DvH (see *Materials and methods* and Supplementary Table S1). As expected, this thermodynamic view shows the feasibility of lactate oxidation in the presence of sulfate, where electrons can flow from the lactate dehydrogenase (LDH) catalyzed reaction to sulfate reduction reactions, in accordance with the standard reduction potentials of these reactions (Figure 4). This would allow DvH to harvest the available energy for growth. In the absence of sulfate as a terminal electron acceptor, however, lactate oxidation would only be possible if the LDH-catalyzed reaction could be coupled with an appropriate electron accepting reaction. Suitable reactions for this role, and available in the central metabolism of DvH, would include the reduction of acetaldehyde to ethanol and reduction of pyruvate to alanine. Combining the lactate oxidation with either of these reactions would lead to a thermodynamically feasible pathway; however, they would result in the by-passing of the ATP generating step of the lactate oxidation pathway; the conversion of pyruvate to acetate mediated by the enzymes pyruvate oxidoreductase, phosphate acetyltransferase and acetate kinase (POR-PTA-ACK in Figure 4). A third option as an electron accepting reaction would be the hydrogenase-catalyzed proton reduction, leading to hydrogen formation (Walker *et al.*, 2009) (Figure 4). Coupling this reaction with lactate oxidation would allow production of pyruvate, with subsequent pyruvate oxidation via POR-PTA-ACK pathway leading to ATP formation.

The free energy of the combined lactate oxidation–proton reduction reaction under standard conditions is, however, strongly positive (43.2 kJ mol^−1^), creating an uphill energy barrier for growth (see *Materials and methods*, Supplementary Table S1). Changing this energy budget in order to make this fermentative reaction thermodynamically feasible would require reducing the hydrogen and pyruvate concentrations. In particular, assuming biologically realistic concentrations (Bennett *et al.*, 2009) of 1 mmol and 0.1 mmol for lactate and pyruvate respectively, then the hydrogen partial pressure (that is, concentration) required to achieve a negative reaction free energy would be around 0.03Pa. This pressure is well below the thermodynamically allowed minimum hydrogen pressure of 0.2Pa required for hydrogenotrophic methanogenesis (Schink, 1997). Thus, at the possible hydrogen pressures that can lead to syntrophy, the fermentative lactate oxidation metabolism might stall due to thermodynamic limitations. One way to overcome this thermodynamic limitation would be to invest additional energy into the lactate oxidation reaction, with the side effect that a higher hydrogen pressure would result under physiological conditions. As the Coo is annotated as an H^+^/Na^+^ ion-translocating hydrogenase, we hypothesize that the identified polymorphism in this gene increases the number of ions it can translocate over the membrane per number of hydrogen produced, and thereby use the membrane gradient as a form of cellular energy to invest in lactate oxidation. In particular, each ion translocated per hydrogen produced could result up to a hundred-fold increase in equilibrium hydrogen pressure tolerated by DvH (that is, from 0.03Pa to 3Pa for single ion, assuming 2 pH units membrane gradient). In a view based on electron flow, this scenario could be seen as shifting the reduction potential of the Coo-catalyzed proton reduction reaction to more positive values, and thereby making it thermodynamically feasible for this reaction to accept electrons from LDH-mediated lactate oxidation reaction (Figure 4). We also note that the function of hydrogenases, and in particular their ability to shuttle hydrogen and protons to the reaction center, commonly involves selenocysteine and cysteine residues (Valente *et al.*, 2005; Arnér, 2010). As such, it is tempting to speculate that the mutation observed here in the *cooK* gene involves the replacement of a selenocysteine residue with serine in the *cooK* gene product, with direct effect on enzyme kinetics or reduction potential of the catalyzed proton reduction, as seen in some hydrogenases (Arnér, 2010; Gutiérrez-Sánchez *et al.*, 2010). A sequence analysis shows that many SRBs contain a homolog of the CooK subunit and carry a conserved cysteine residue in the identified loci (Supplementary File 2).

### Physiology of DvH-s is in line with the expectations of the thermodynamic model

**Figure 5:**
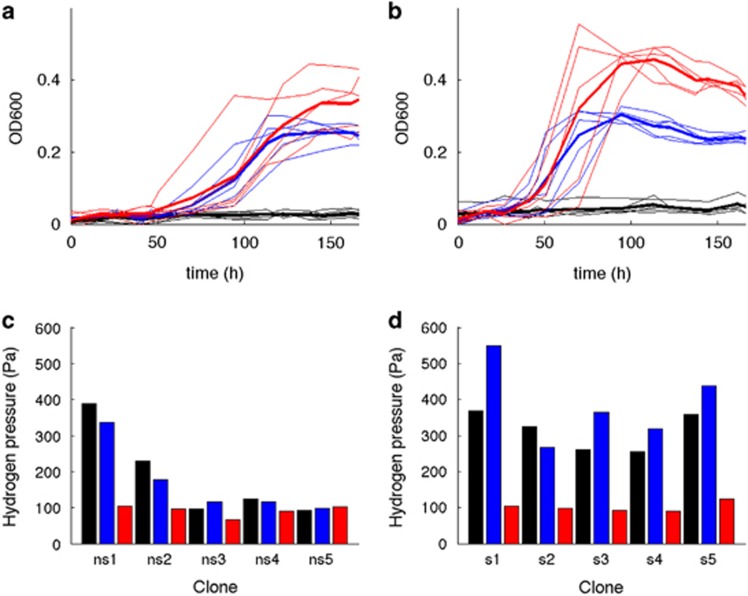
OD600 (**a** and **b**, thick lines are mean of five thin lines) and cumulative hydrogen pressure (**c** and **d**) after 168 h of incubation for isolated DvH-ns (**a** and **c**) and DvH-s (**b** and **d**) clones. Cultures were grown on CCM containing 30 mm lactate and varying amounts of SO_4_^2−^. Black: 0 mm SO_4_^2−^, blue: 7.5 mm SO_4_^2−^, red: 15 mm SO_4_^2−^ (see *Materials and methods*). Background (ambient) hydrogen pressure was 90 Pa.

A key testable prediction stemming from the thermodynamic model is that DvH-s (as well as DvH-ns 1 and 2) clones should produce more hydrogen under sulfate-limited conditions. We verified this prediction by inoculating all 10 sequenced clones on a lactate minimal medium without sulfate, and with 50% and 100% of the sulfate needed to degrade all available lactate via the sulfate pathway (see *Materials and methods*). We found that all DvH-s clones, as well as DvH-ns 1 and 2 clones, produce significant hydrogen above background levels in the absence of sulfate or at 50% sulfate (*P*⩽0.01, two-tailed *t*-test), while DvH-ns 3, 4 and 5 clones only produce hydrogen significantly above background levels at 50% sulfate (*P*⩽0.05, two-tailed *t*-test) (Figure 5). The cumulative hydrogen pressure achieved by the former clones over the course of 7 days, in the absence of sulfate and at stationary state is ~300 Pa, well above the pressure needed to sustain Mm growth. This explains the ability of the DvH-s clones to engage in syntrophy with Mm.

**Figure 6:**
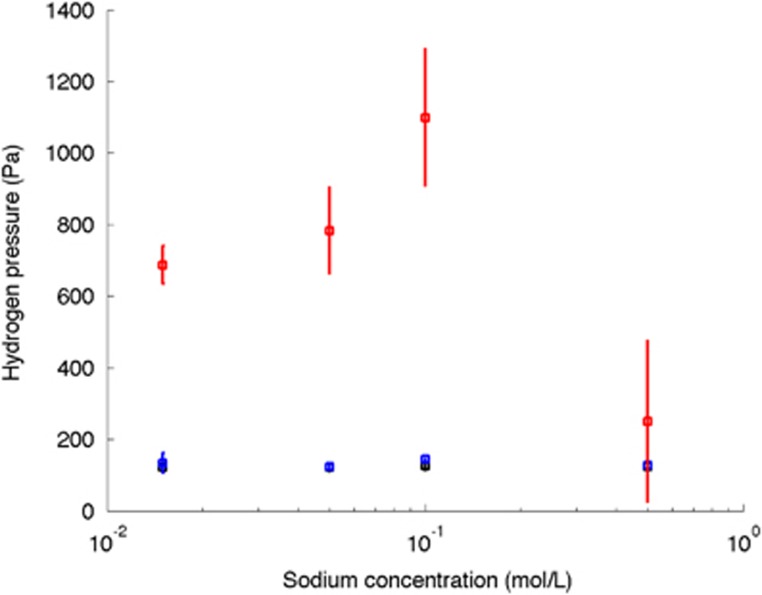
Mean hydrogen pressure and standard deviation of triplicate measurements of DvH-s (red), DvH-ns (blue) and blank medium (black) at different concentrations of sodium. Headspace measurements were performed after 6 days of incubation without detectable growth in the Na-Buffer (see *Materials and methods*). A full color version of this figure is available at the *The ISMEJ Journal* journal online.

A second prediction stemming from the thermodynamic model discussed above is that hydrogen production of syntrophic DvH-s clones should be affected by modifications in the ion content of the media. The observed cumulative hydrogen pressure of ~300Pa from DvH-s clones shows that indeed there is an energy investment into the lactate oxidation within this genotype. Dependent on physiological equilibrium concentrations of lactate, pyruvate and the magnitude of the ion gradient across the membrane, this investment could be achieved by Coo translocating as low as 1 H^+^/Na^+^ per hydrogen produced (in Figure 4 we show that this investment needs to be ~−23 kJ mol^−1^, which under standard conditions equates to translating 2 single charged ions over a 100-fold ion gradient per hydrogen produced). At any rate, changing the Na^+^ concentration in the media should affect the membrane ion gradient, and thereby the level of energy investment and the steady-state hydrogen pressure. To test this, we transferred triplicates of DvH-s and DvH-ns cultures into Na-buffer at different Na^+^ concentrations, and after 6 days we measured the equilibrium hydrogen pressure. Although these cultures did not show any detectable growth, the DvH-s clones produced consistently more hydrogen than the DvH-ns clones, and such production increased with increasing Na^+^ concentration up to a threshold high concentration (Figure 6). The low hydrogen production at 500 mm Na^+^ indicates that at that level of Na^+^ concentration the salinity tolerance of DvH is exceeded (Zhou *et al.*, 2013).

## Conclusions

It is well known that SRBs can act as both hydrogen consumers and producers, taking the latter role in the absence of terminal electron acceptors such as sulfate (Muyzer and Stams, 2008). This is believed to underpin the ability of SRBs to occupy different niches and survive different environmental conditions (Muyzer and Stams, 2008). Our findings suggest that the dual physiology is encoded in DvH as two separate genotypes, which are maintained at different frequencies. As indicated by theoretical studies of phenotypic variability under fluctuating environments (Kussell and Leibler, 2005), it is possible that these DvH genotype frequencies are in alignment with the frequency of the fluctuations in the environment in terms of availability of strong electron acceptors such as sulfate. At the same time, it is also clear that the frequency at which the syntrophic DvH genotype is maintained will be directly linked to the presence of syntrophic partners such as Mm in the environment. It remains to be seen if a stable polymorphism as seen in DvH is a strategy common to other SRBs in the environment. From a more applied point, this study points to engineering of thermodynamic constraints in metabolism as a possible route to the implementation of syntrophic interactions in synthetic microbial communities.
