# Supplementary material for: A stable genetic polymorphism underpinning microbial syntrophy
Source: ISME J. 2016 Jun 3;10(12):2844–53. doi: 10.1038/ismej.2016.80 (PMC5042321; doi:10.1038/ismej.2016.80)
Supplement: Supplementary Methods [file ismej201680x1.docx]

**A stable genetic polymorphism underpinning microbial syntrophy**

**Tobias Großkopf^1*^, Simone Zenobi^1^, Mark Alston^2^, Leighton Folkes^2^, David Swarbreck^2^, Orkun S Soyer^1*^**

**Supplementary Information**

1. Detailed recipes of the media used during the study and protocol for preparation of anaerobic media
2. Procedure for OD measurements
3. Procedure for Gas (Methane & hydrogen) measurements
4. Supplementary Figure 1: Schematic of experimental setup

**1. Detailed recipes of the media used during the study and protocol for preparation of anaerobic media**

**1.1 Detailed recipes of the media used during the study**

***M141 medium, used for M. maripaludis monoculture***

Basal salt mix, per liter: KCl: 0.34g, MgCl_2_ x 6H_2_O: 4g, MgSO_4_ x 7H_2_O: 3.45g, NH_4_Cl: 0.25g, CaCl_2_ x 2H_2_O: 0.14g, K_2_HPO_4_: 0.14g, NaCl: 18g, Fe(NH_4_)_2_(SO_4_)_2_ x 7H_2_O: 2mg, NaHCO_3_: 1g, Yeast extract (Difco) 2g, Trypticase (BBL): 2g.

Trace element solution (100 X): In 850 mL of dH_2_O, dissolve 1.5g Nitrilotriacetic acid and adjust pH to 6.5 with KOH. Then, add: MgSO_4_ x 7H_2_O: 3g, MnSO_4_ x H_2_O: 0.5g, NaCl: 1g, FeSO_4_ x 7H_2_O: 0.1g, CoSO_4_ x 7H_2_O: 0.18g, CaCl_2_ x 2H_2_O: 0.1g, ZnSO_4_ x 7H_2_O: 0.18g, CuSO_4_ x 5H_2_O: 0.01g, KAl(SO_4_)_2_ x 12H_2_O: 0.02g, H_3_BO_3_: 0.01g, Na_2_MoO_4_ x 2H_2_O: 0.01g, NiCl_2_ x 6H_2_O: 0.03g, Na_2_SeO_3_ x 5H_2_O: 0.3mg. Bring final volume to 1L with dH_2_O. Final pH is adjusted to 7 with HCl and NaOH.

Vitamin solution (100X): In 1L of dH_2_O, dissolve: Biotin: 2mg, Folic acid: 2mg, Pyridoxin-HCl: 10mg, Thiamine-HCl x 2H_2_O: 5mg, Riboflavin: 5mg, Nicotinic acid: 5mg, Vitamin B12: 0.1mg, p-Aminobenzoic acid: 5mg, Lipoic acid: 5mg. Vitamins were filter sterilized into a sterile anaerobic serum flask (30 mL in 50 mL flask), crimp sealed and degassed by flushing the headspace of the vial for 30 minutes with oxygen free nitrogen at a flow rate of 0.5 LPM through blue cannulas (0.6 mm ID, Microlance, Beckton Dickinson, Franklin Lakes, NJ, USA) equipped with a sterile filter (Minisart, Sartorius, Göttingen, Germany) on the gassing line.

***Postgate Medium C, used for D. vulgaris monoculture (Postgate, 1984)***

Basal salt mix, per liter: K_2_HPO_4_: 0.5g, NH_4_Cl: 1g, Na_2_SO_4_: 4.5g, CaCl_2_ x 6H_2_O: 0.06g, MgSO_4_ x 7H_2_O: 0.06g, Na-Lactate: 3.36g, Yeast extract: 1g, FeSO_4_ x 7H_2_O: 5 mL stock (0.19g FeSO_4_ x 7H_2_O in 100mL 10mM HCl), Na_3_-Citrate x 2H_2_O: 0.3g.

For plating, 15 g Agar where added to 1L of medium. The medium was autoclaved and transferred to the anaerobic chamber while the agar was still liquid, where plates were poured and allowed to degas for 12 hours before *D.vulgaris* was streaked onto the plates from liquid culture (50 µL). Ten fold dilution series of cultures with growth medium where prepared from neat to 10^-6^ and the plates showing single colony growth used for clone picking.

***Co-Culture medium (CCM) (modified from Walker et al. 2009), used for D.vulgaris – M.maripaludis co-culture***

Basal salt mix: In 1L dH_2_O, dissolve: K_2_HPO_4_: 0.19g, NaCl: 2.17g, MgCl_2_ x 6H_2_O: 5.5g, CaCl_2_ x 2H_2_O: 0.14g, NH_4_Cl: 0.5g, KCl: 0.335g, NaHCO_3_: 2.5g, Na-Lactate: 3.36g. Sulfate was added for experiments as mentioned in the text as Na_2_SO_4_: 1.065g (7.5 mM) or 2.13g (15 mM) to achieve the desired sulfate concentration.

Trace element solution (100X): In 850 mL of dH2O, dissolve 1.5g Nitrilotriacetic acid and adjust the pH to 6.5 with KOH. Then add: MgCl_2_ x 6H_2_O: 2.48g, MnCl_2_ x 4 H_2_O: 0.585g, NaCl: 1g, FeCl_2_ x 4 H_2_O: 0.072g, CoCl_2_ x 6 H_2_O: 0.152g, CaCl_2_ x 2 H_2_O: 0.1g, ZnCl_2_ x 4 H_2_O: 0.085g, CuCl_2_: 0.005g, AlCl_3_: 0.01g, H_3_BO_3_: 0.01g, Na_2_MoO_4_ x 2 H_2_O: 0.01g, NiCl_2_ x 6 H_2_O: 0.03g, Na_2_SeO_3_ x 5 H_2_O: 0.0003g, Na_2_WO_4_ x 2 H_2_O: 0.008g. Bring final volume to 1L with d H_2_O. Final pH is adjusted to 7 with HCl and NaOH.

Vitamin solution (100X): like for M141.

***Na-Buffer, used for experiments with D. vulgaris monocultures***

Basal salt mix: In 1L dH_2_O, dissolve: KH_2_PO_4_: 0.5g, NH_4_Cl: 1g, CaCl_2_ x 2H_2_O: 0.04g, MgCl_2_ x 6H_2_O: 0.05g, Na-Lactate: 1.12g. Na_3_-Citrate x H_2_O: 0.3g, FeSO_4_: 5mL of a 1,9g/L stock solution. The resulting medium has a Na^+^ concentration of 15 mM. To generate buffer with Na^+^ concentrations of 50, 100 and 500 mM, 0.409g, 0.994g and 5.669g of NaCl where added, respectively.

**1.2. Protocol for preparation of anaerobic media**

All media are prepared by mixing the basal salt solution and adding 10 mL of the trace element solution (except for Postgate medium C, Na-Buffer) and 1 mL Resazurin stock (1g/L) to 1L. M141, CCM and Na-Buffer are adjusted to pH 7, while the Postgate medium C is adjusted to pH 7.2 with 1N HCl or NaOH. Then 200 mL of the medium is brought to the boiling point in a 500 mL conical flask while a continuous flow of anoxic gas (100% N_2_ for medium C, 80% N_2_ + 20% CO_2_ for M141 and CCM) is maintained at 0.5 LPM flow rate into the headspace of the flask with a cannula (using a rubber stopper to close off the top opening). After boiling initiates, the heating of the medium is stopped and the medium left to cool down for 1 hour while stirring at medium speed and maintaining the gas flow as above. For M141 and CCM, vitamins are added (2mL/ 200mL) into each after boiling, when the medium has cooled to 50°C. Further, 2mL/200mL or 3mL/200mL of an anoxic Cysteine-HCl stock (0.2M) is added to M141 and CCM, respectively. The removal of oxygen is verified by a color-shift from pink to colorless by the Resazurin. The medium is quickly moved to an anaerobic chamber (MACS-MG-500 anaerobic workstation, Don Whitley Scientific, Shipley, UK) and the medium filled in 5 mL aliquots into 27 mL Balch tubes (Chemglass Life Sciences, Vineland, NJ, USA) that had been degassed for 24 hours in the anaerobe chamber. Tubes are closed with blue butyl rubber septa (Chemglass Life Sciences, Vineland, NJ, USA) and crimp sealed. Next, tubes are autoclaved for 15 minutes at 121°C in a desktop autoclave (ST 19 T, Dixon, Wickford, UK). Before inoculation with culture, 50 µL of an anaerobic 0.1 M Na_2_S x 9H_2_O stock is added to 5mL of medium. Monocultures of *M.maripaludis* are treated after inoculation in the following way: The headspace of the anaerobe tube is flushed for 1 minute with sterile H_2_/CO_2_ (80%/20%) gas at a flow rate of 0.5LPM. Then the outgassing needle is removed and the pressure equilibrated to 2 bar with the same gas mixture. All gases used for headspace flushing are run through an oxygen scrubber column (Oxisorb, MG Industries, Bad Soden, Germany), to remove any residual oxygen. All chemicals used are analytic grade or higher (>= 98% purity, Sigma-Aldrich, St. Louis, MO, USA).

**2. Optical density measurements**

Optical density at 600 nm (OD600) was measured in anaerobic tubes in a Spectronic 200 photometer fitted with test-tube holder (Thermo Scientific, Waltham, MA, USA). Un-inoculated medium from the same batch was used as blank, and raw reads where corrected for the anaerobe tube thickness to result in a 1 cm path length according to equation 1.

 (*Eq.1*)

**3. Gas production measurements**

Headspace pressure of sealed incubation vessels (anaerobic tubes or serum flasks) was measured with a needle pressure gauge (Ashcroft, Stratford, CT, USA) by pinching twice through the septum with blue needles of 0.6 mm inner diameter (Microlance, Becton Dickinson, Franklin Lakes, NJ, USA) and recording both reads as *p_1_* and *p_2_*. The total headspace pressure (atm) was calculated correcting for the instruments dead volume, according to equation 2.

 (*Eq.2*)

After pressure measurements, headspace pressure was equilibrated to 1 atm by allowing the overpressure within the vessel to remove the piston of a glass syringe until equilibration. Next, 100 µL of headspace where taken into a 100 µL glass syringe fitted with pressure lock (Pressure-Lok, Vici, Baton Rouge, LA, USA) through the septum. The pressure lock was closed and the needle changed for a blunt end needle for injection into a gas chromatograph. Next, the 100 µL gas was injected into an Agilent 7890A (Agilent Technologies, Craven Arms, UK) equipped with a 6 foot 60/80 mole sieve 5A column (Supelco, Gillingham, UK) for hydrogen measurements. For methane measurements, the 100 µL were injected into an Agilent 7890A equipped with a 6 foot 60/80 mole sieve 5A column and a 6 foot 80/100 Porapak Q column (Supelco, Gillingham, UK). A 99.995% - pure methane (Thames-Restec, Saunderton, UK) or 80%/20% H_2_/CO_2_ mixture (BOC, Coventry, UK) were used as standard gases for calibration of methane and hydrogen measurements, respectively. After confirming linearity of measurements from 0-100% (methane) (r^2^=0.998) and 0-80% (H_2_) (r^2^=0.993) by running a five point calibration curve, conversion factors for peak area to gas concentration where achieved by a two point calibration using triplicate standards and triplicate blanks (100% N_2_ gas) on each measurement day. The fraction of gas in the injected sample was calculated according to equation 3:

 (*Eq.3*)

where *f_gas_* and *f_std_* are the fraction of methane or hydrogen in the sample headspace and the standard, respectively, and *m_sample_*, *m_blank_* and *m_std_* are the measured peak areas (methane detection) or peak heights (hydrogen detection) of the sample, the blank and the standard, respectively. The total gas in the sample vial is calculated, using the pressure from equation 2 and the gas fraction from equation 3:

 (*Eq.4*)

where *v_gas_* and *v_headspace_* are the volume of gas under consideration (methane or hydrogen) and the headspace volume of the vial respectively, and *p_atm_* is atmospheric pressure.

**4. Supplementary Figure 1**

**
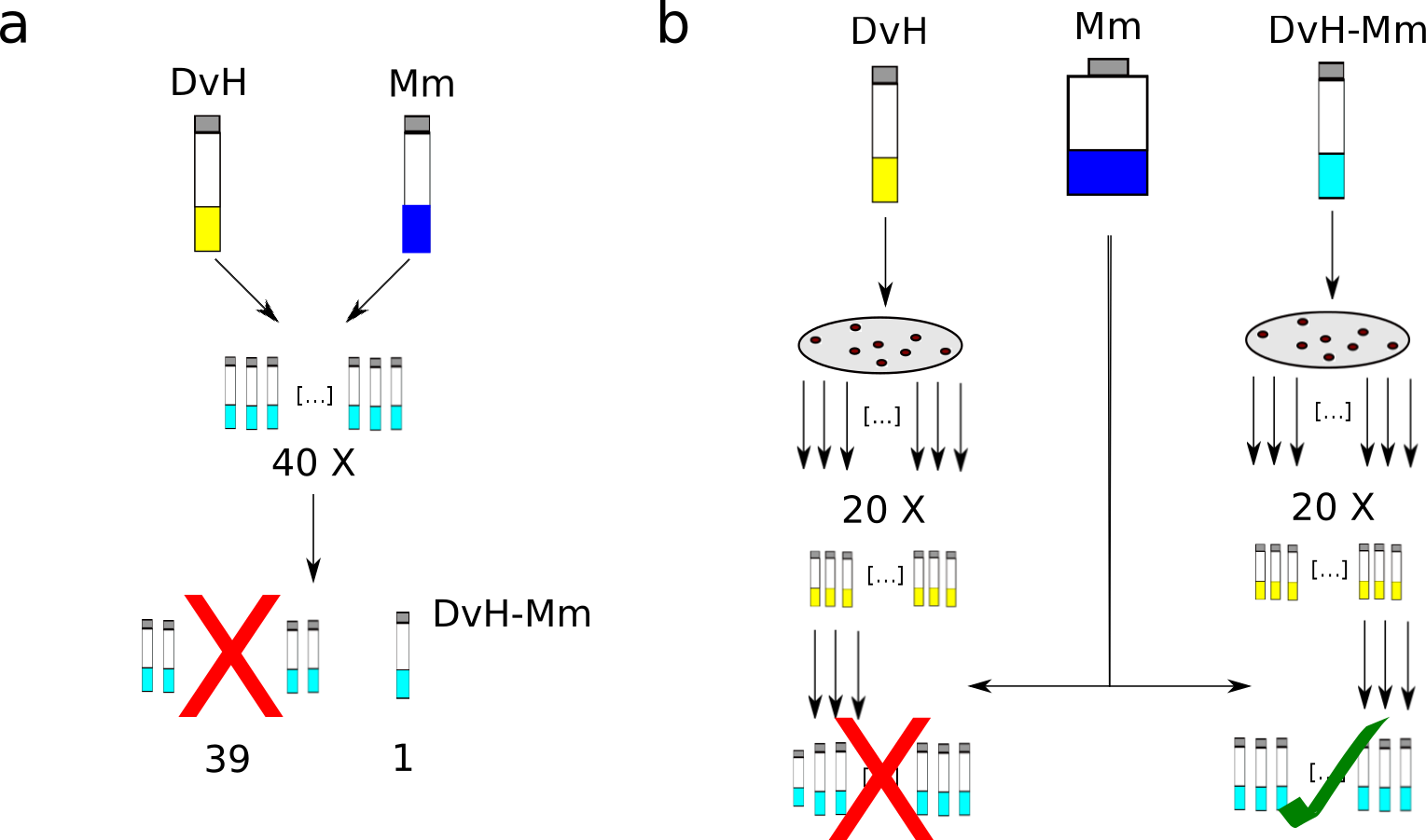
**

**Figure S1.** Schematic of the experimental setup lead to the identification of DvH-ns and DvH-s clones. For details, see main text.

**References**

Postgate JR. (1984). The sulfate reducing bacteria. 2nd ed. Cambridge University Press: Cambridge.

Walker CB, He Z, Yang ZK, Ringbauer J a, He Q, Zhou J, *et al.* (2009). The electron transfer system of syntrophically grown *Desulfovibrio vulgaris*. *J Bacteriol* **191**: 5793–801.
