# Supplementary material for: A stable genetic polymorphism underpinning microbial syntrophy
Source: ISME J. 2016 Jun 3;10(12):2844–53. doi: 10.1038/ismej.2016.80 (PMC5042321; doi:10.1038/ismej.2016.80)
Supplement: Supplementary File 2 [file ismej201680x3.pdf]

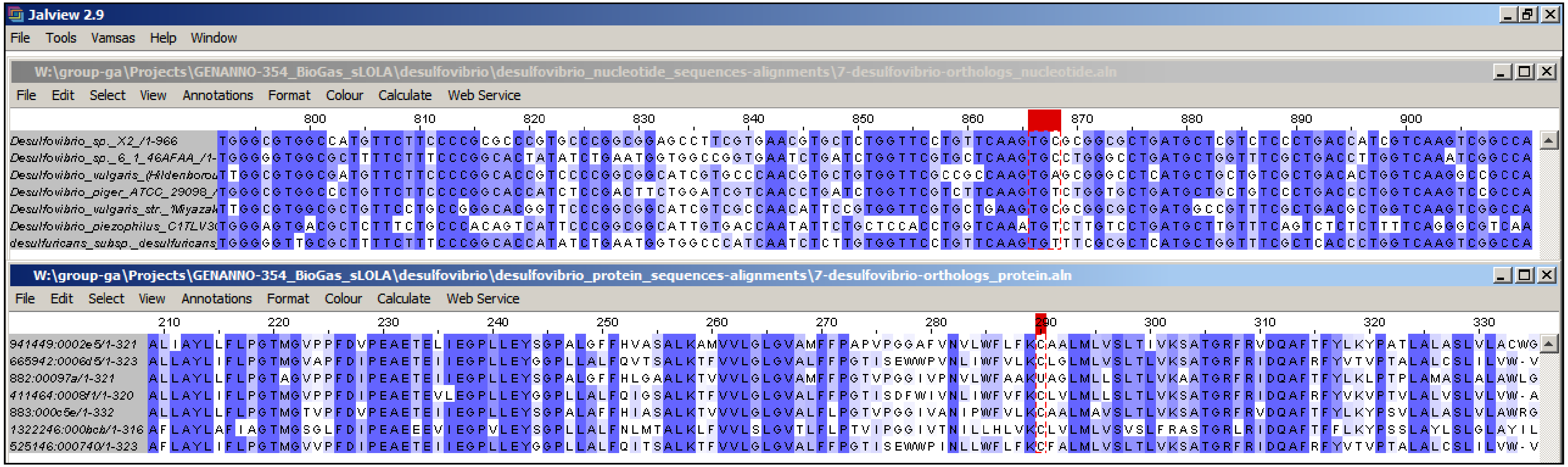

Nucleotide and protein alignments for Desulfovibrio orthologs to DVU_2287 [Desulfovibrio vulgaris (Hildenborough)]
Aligned via JalView using TCoffee [defaults], coloured by ‘percentage identity’.
The ‘U’ residue/codon is in the red-highlighted box.

Orthologs aligned by organism [top-to-bottom]:

Desulfovibrio sp. X2
Desulfovibrio sp. 6_1_46AFAA
Desulfovibrio vulgaris (Hildenborough)
Desulfovibrio piger ATCC 29098
Desulfovibrio vulgaris str. 'Miyazaki F'
Desulfovibrio piezophilus C1TLV30
Desulfovibrio desulfuricans subsp. desulfuricans str. ATCC 27774

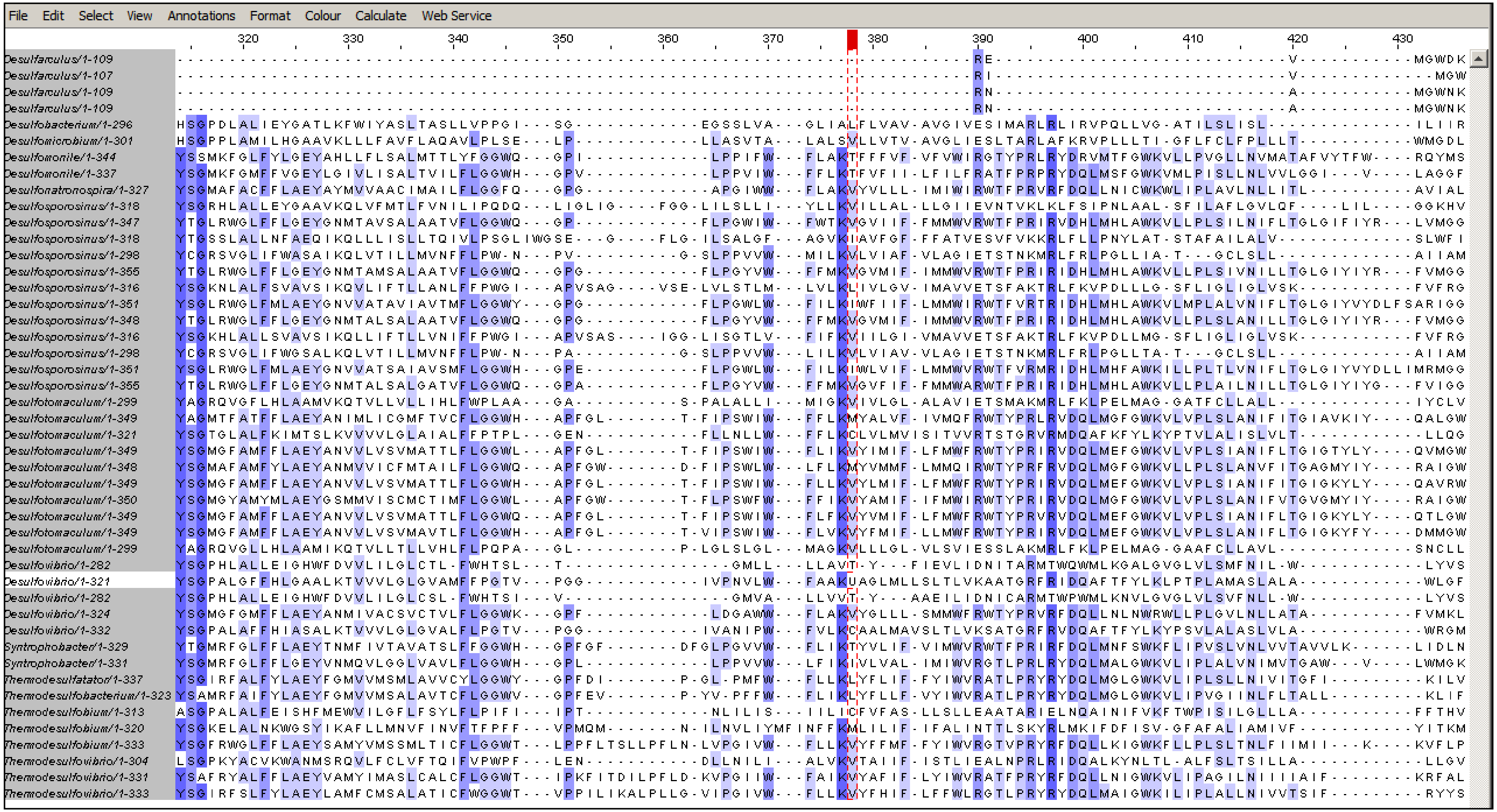

Protein alignments for sulphate-reducing bacteria orthologs to DVU_2287 [Desulfovibrio vulgaris (Hildenborough); row highlighted in white]. Some species have more than one ortholog.

Aligned via JalView using TCoffee [defaults], coloured by 'percentage identity'. The 'U' residue is in the red-highlighted column.

Orthologs aligned by organism [top-to-bottom]:
